# Global Research Trends in Biomimetic Lattice Structures for Energy Absorption and Deformation: A Bibliometric Analysis (2020–2025)

**DOI:** 10.3390/biomimetics10070477

**Published:** 2025-07-19

**Authors:** Sunny Narayan, Brahim Menacer, Muhammad Usman Kaisan, Joseph Samuel, Moaz Al-Lehaibi, Faisal O. Mahroogi, Víctor Tuninetti

**Affiliations:** 1 Department of Mechanics and Advanced Materials, Campus Monterrey, School of Engineering and Sciences, Tecnológico de Monterrey, Av. Eugenio Garza Sada 2501 Sur, Tecnológico, Monterrey 64849, Mexico; 2 Laboratoire des Systèmes Complexes (LSC), Ecole Supérieure en Génie Electrique et Energétique ESGEE Oran, Chemin Vicinal N9, Oran 31000, Algeria; acer.msn@hotmail.fr; 3 Mechanical Engineering Department, Ahmadu Bello University, Kaduna, Zaria 810107, Nigeria; engineer.kaisan@gmail.com; 4 Department of Mechanical Engineering, Baze University, Abuja 900108, Nigeria; samuel.joseph@bazeuniversity.edu.ng; 5 Department of Mechanical Engineering, College of Engineering and Architecture, Umm Al-Qura University, P.O. Box 5555, Makkah 24382, Saudi Arabia; molehaibi@uqu.edu.sa; 6 Department of Mechanical Engineering, Islamic University of Madinah, Madinah 42351, Saudi Arabia; f.mahroogi@iu.edu.sa; 7 Mechanical Engineering Department, Universidad de La Frontera, Temuco 4811230, Chile; victor.tuninetti@ufrontera.cl

**Keywords:** biomimetic lattices, lattice structures, structural optimization, lattice mechanics, energy absorption, computational modeling, smart materials

## Abstract

Biomimetic lattice structures, inspired by natural architectures such as bone, coral, mollusk shells, and Euplectella aspergillum, have gained increasing attention for their exceptional strength-to-weight ratios, energy absorption, and deformation control. These properties make them ideal for advanced engineering applications in aerospace, biomedical devices, and structural impact protection. This study presents a comprehensive bibliometric analysis of global research on biomimetic lattice structures published between 2020 and 2025, aiming to identify thematic trends, collaboration patterns, and underexplored areas. A curated dataset of 3685 publications was extracted from databases like PubMed, Dimensions, Scopus, IEEE, Google Scholar, and Science Direct and merged together. After the removal of duplication and cleaning, about 2226 full research articles selected for the bibliometric analysis excluding review works, conference papers, book chapters, and notes using Cite space, VOS viewer version 1.6.20, and Bibliometrix R packages (4.5. 64-bit) for mapping co-authorship networks, institutional affiliations, keyword co-occurrence, and citation relationships. A significant increase in the number of publications was found over the past year, reflecting growing interest in this area. The results identify China as the most prolific contributor, with substantial institutional support and active collaboration networks, especially with European research groups. Key research focuses include additive manufacturing, finite element modeling, machine learning-based design optimization, and the performance evaluation of bioinspired geometries. Notably, the integration of artificial intelligence into structural modeling is accelerating a shift toward data-driven design frameworks. However, gaps remain in geometric modeling standardization, fatigue behavior analysis, and the real-world validation of lattice structures under complex loading conditions. This study provides a strategic overview of current research directions and offers guidance for future interdisciplinary exploration. The insights are intended to support researchers and practitioners in advancing next-generation biomimetic materials with superior mechanical performance and application-specific adaptability.

## 1. Introduction

Lattice structures are fundamental units for understanding the design, construction, nature, and behavior of material structures. Although lattice structures, like metamaterials, are based on repeating unit cells, they differ from natural crystal lattices in origin and design. Each point of a crystal lattice represents the position of an ion, atom, or molecule; however, lattice structures are engineered materials designed to exhibit properties not found in natural materials. According to Gibson and Ashby Evans [1], they can be classified as “cellular structures,” which include honeycombs, lattices, and foams. Specifically, a lattice structure is a cellular structure composed of a porous material organized in space in the form of a periodic lattice. Lattice structures are characterized by an interconnected network of edges and faces [2] and can be closed-cell with isolated pores or open-cell with interconnected pores.

The structural efficiency observed in natural formations such as trabecular bone, fish scales, and honeycombs has motivated engineers to create biomimetic lattice structures that possess high strength-to-weight ratios and energy absorption capabilities. Metals such as Ti 6Al 4V demonstrate high stiffness but exhibit lower impact absorption, whereas polymer-based lattices like TPU provide enhanced flexibility and resilience when subjected to impact loading. The design of various natural structures, including trabecular bone [3], fish scales [4], spider webs [5], honeycombs [6], mollusk shells [7], and animal horns [8], has inspired engineers for centuries to create efficient and durable structures.

The biomimetic design of these lattice structures presents a distinctive combination of versatile properties such as lightweight, mechanical resilience, and energy absorption, rendering them appealing for applications in architecture [9], aerospace [10,11], additive manufacturing [12,13], automotive [14], marine [15], and biomedical fields [16]. A biphasic biomimetic bone scaffold has been developed through the bidirectional regulation of endochondral ossification [17]. The European Aeronautic and Defense Agency optimized a hinge for an Airbus A320 utilizing biomimetic design concepts [18]. NASA is investigating the application of electroactive polymers as artificial muscles, which play a crucial role in the advancement of biologically inspired technologies [19].

Considering the fundamental research on lattice structures, the subsequent section examines the latest developments in assessing their deformation properties through both experimental and computational methods. To begin with, we conducted research and gathered information regarding highly regarded journals and frequently cited articles pertaining to the overarching subject of lattice structures (Table 1).

Verma et al. [23] conducted a study on the removal of polymer powder-based additive manufacturing during the process. They calculated the total volumetric energy absorbed, energy efficiency of absorption, and the onset of strain for various 3D-printed lattice structures subjected to compression [24]. The dual-lattice exhibited a greater energy-absorbing capacity at each volume fraction. A lotus root lattice structure (LRLS) was proposed and fabricated using 3D printing technology [25]. The finite element analysis of LRLS has demonstrated its effectiveness. Additionally, an octagonal lattice structure produced through additive manufacturing has been suggested [26]. The applicability of a Kelvin lattice structure has been explored for its potential use as a bone scaffold [27]. An optimization model was introduced for designing the cooling channel in gas turbine blades [28]. Relationships between heat transfer coefficients were established between a pyramid-type lattice structure (PLS) and a Kagome-type lattice [28]. Xinwei et al. [29] employed resonance and Bragg’s scattering to assess the acoustic performance of hybrid lattice structures, discovering that these properties were influenced by the size of the cells. Palomba et al. [30] examined bamboo samples through impact testing and found that the softer layers of the material exhibited a higher energy absorption capacity. Furthermore, an investigation into the use of lattice structures in marine shipbuilding has been conducted. Table 2 provides a summary of the reviewed papers detailing specific applications of biomimetics.

There are many obstacles to the widespread commercial application of these structures, primarily due to challenges in manufacturing and the unpredictable nature of fatigue behavior. The majority of the current research has concentrated on existing cellular structures without altering the topography of the cells or taking into account the Poisson ratio. Further investigation is required into the effects of layer design and load-bearing capacities on orientation.

A bibliometric analysis (BA) of lattice structure materials covering the period from 2002 to 2022 was conducted [33]. A systematic review of 2512 journal articles sourced from the Scopus database revealed a significant bias towards the study of materials [34]. The Bibliometrix package, applied to 1271 documents from Scopus, indicated a growth rate of 20.8% over the years, with the United States leading in publications, followed by China and the United Kingdom [35]. Similar findings were observed in recent studies. Research on additive manufacturing for medical applications was most prevalent in the years 2010 and 2012. According to the reviews, there are 2769 pieces of literature related to the theme of 3D printing [36]. An analysis of keyword frequency revealed that the selective laser melting and fused deposition modeling techniques were the two most frequently studied, accounting for 35.76% and 20.09%, respectively [37]. A bibliometric analysis of fused deposition modeling (FDM) was performed to gain insights into the trends and research areas [38].

The landing of the aluminum strut-based Team Indus spacecraft on the Moon in 2020 signified the beginning of a new era in the application of lightweight and structurally sound lattice structures [39]. A groundbreaking 3D acoustic black hole (ABH) was employed to attain high load-bearing capacity and vibration suppression [40]. Recently, topology optimization and data-driven techniques have been utilized [41]. Several challenges, such as computational complexity, material synthesis, and interdisciplinary collaboration, have been recognized in the application of these structures [42]. These findings suggest that the designated timeframe (2020–2025) is critical or strategic for the current study.

As shown in Table 1, previous studies have examined various specific elements of these structures; however, there is a lack of a thorough review of recent advancements in these structures designed to emulate biomimetic applications with respect to energy and deformation. Therefore, a comprehensive bibliometric review has been provided based on these criteria. The primary objectives of this analysis are as follows:

- Research Question 1 (RQ1). What materials, methods, and thematic areas are most frequently explored in the recent literature on biomimetic lattice structures?
- Research Question 2 (RQ2). How has the volume of scientific publications evolved between 2020 and 2025 in relation to deformation and energy absorption in biomimetic lattice design?
- Research Question 3 (RQ3). Who are the most influential authors, journals, and countries contributing to this research domain?
- Research Question 4 (RQ4). What are the key research gaps and emerging themes that require further investigation in the field?

The primary aim of this work is to offer insights into the most notable contributions and collaborations in the field of lattice structure design over the last five years, as well as to explore the key topics and scholarly articles. This study holds significance as safe design is crucial for maintaining structural integrity. The manuscript is divided into six sections. In Section 2, we have presented an overview of techniques, methods, and materials for designing these structures. The process of bibliometric analysis is presented in Section 3. We have tried to identify the most active keywords, journals, researchers, their institutions, collaborations, and countries in Section 4, which could be advantageous for the relevant scientific community. Additionally, we examined the emergence of new themes that may inspire researchers to explore lesser-known areas in Section 5. By providing insights into ongoing research and future directions in Section 6, this review could serve as a vital resource for various industries.

## 2. Exploring Recent Advances in Materials, Methods, and Thematic Areas of Biomimetic Lattice Structures Research

In this section, we aim to investigate the answers to RQ1. Lattice structures subjected to shock and impact loading can undergo deformation, resulting in the propagation of shock waves [27]. The findings indicated that the elastic modulus of Silicon Carbon scaffolds was the lowest, while that of Silicon Iron Carbon scaffolds was the highest. McKown et al. conducted experimental evaluations of the response and dynamic behavior of steel lattice structures when exposed to impulsive loads and their corresponding failure modes [43]. It was observed that the resistance of lattice structures to blasts increased with higher yield strength. The impact response of lattice structures at elevated deformation rates was assessed using Hopkinson bar tests. Materials like foams, glass, ceramic, or any biological materials may be used for this test. A standard setup comprises two elongated bars aligned along their axes. Both bars are constructed from the same material and feature a circular cross-section; the specimen is secured between the two bars. A striker, propelled by a gas gun, strikes the first (incident) bar, generating a pressure pulse. This pressure wave traverses the incident bar, where it is partially reflected and partially transmitted to the second (transmission) bar at the specimen. The wave signal was captured by strain gauges positioned at the center of both bars. These tests were performed on four composite samples of graphene-based nanoplates (GNPs) with stress–strain plots [44]. The armature used in DC machines for this test has four slots of 1 mm in size in the armature coil. The size and number of slots affect performance characteristics. In the Hopkinson test, different machines are chosen to be identical for effective comparison of results under the same testing conditions, meaning all machines would have the same number of slots of the same size.

### 2.1. Finite Element Analysis for Deformation Prediction

Finite element analysis (FEA) conducted with Abaqus or any other interface can effectively simulate the deformation behavior of lattice structures. Digital image correlation (DIC) was employed to investigate the convergence at the micro-level through the analysis of cell deformation and strain [45]. The energy absorption per unit volume during densification, denoted as Wd, can be determined by integrating the stress–strain response up to the point of densification strain [46]. Finite element (FE) models of the BCC lattices are utilized to forecast the compressive behavior and deformation localization of filaments [47]. A novel hierarchical circular-cell configuration for a lattice structure has been proposed [48]. This structure demonstrated improved uniformity in stress distribution, enhanced mechanical performance, and increased energy absorption capacity. An integrated energy-based approach was applied to analytically derive the effective properties of the missing rib tetra-chiral lattice structure [46]. Strain and compliance tensors were accurately calculated from FE analyses.

### 2.2. Machine Learning Applications in Lattice Design

In recent times, ML and AI models have been extensively used for designing these structures. A multi-step deformation model for lattice structures (MSLSs) was proposed during compression testing [49]. It was determined that stiffness is influenced by the topology of the lattice and the micro-deformation mode of the struts [50,51]. A vortex lattice method (VLM) was developed to evaluate structural deformation [52]. The mechanical and microstructural analysis of IN718 lattice structures produced through additive manufacturing has been conducted [53]. It was observed that the bending mode predominates. Furthermore, a deep learning-based approach was found to greatly enhance stiffness, strength, and isotropic properties [54,55].

The octet-truss lattice structures fabricated from 316 L stainless steel powder underwent SEM and X-ray tomography examinations. Mechanical three-dimensional lattice transformations within a polymeric photonic crystal were investigated [56]. The deformations of the lattice structures were monitored using optical spectroscopy. Experimental findings revealed that the stiffness, strength, and energy absorption of the specimens increased with rising temperatures. Struts with varying diameters were produced using selective laser melting (SLM) [57]. These samples were examined through micro-computed tomography, and the results indicated significant discrepancies in the diameters of the inscribed and circumscribed cylinders, suggesting an elliptical configuration of the struts [58]. The pressure-driven phase transformation of Ti-TiBw composites under cyclic pressures was studied using X-ray diffraction [59]. The variation in bulk modulus illustrated the material’s resistance to deviatoric stress [60]. A novel methodology employing a deep learning-based 3D Generative Adversarial Network (3DGAN) model was introduced to generate aluminum alloy lattice structures [61].

A hierarchical graph attention network (HIGAT) architecture specifically designed for predicting deformations in 3D lattice structures subjected to load was examined [62]. An artificial neural network algorithm was employed to create a relationship model linking the topology of the lattice structure to Young’s modulus, which was subsequently analyzed and validated [63,64]. A material jetting machine was utilized to fabricate intricate lattice structures that exhibited improved elongation and strength [65]. The design and development of lightweight aerospace lattice structures focused on AI customization to address the changing requirements of the aerospace sector.

### 2.3. Experimental Methods for Energy Absorption Measurements

Lattice structures have excellent energy absorption, making them important in the manufacturing of impact protection systems and vehicle and spacecraft parts. One of the most common methods to record the impact energy is the Hopkinson pressure bar (HPB) test discussed at the beginning of this section. The impact of relative density, material type, load, and fabrication techniques on the mechanical performance of additive manufacturing (AM) produced lattice structures was examined [66]. The selection of the stress concentration factor was based on fatigue resistance and S–N curves [67,68]. BCC and BCCZ were created using selective laser melting technology [69,70]. Machine learning models were employed to evaluate the influence of post-processing methods, such as stress relieving and heat treatment, on surface roughness and micro-Vickers hardness [71,72]. The dynamic crashing behavior of innovative bionic honeycombs revealed two distinct deformation phases: initial collapse and subsequent crushing [73,74]. A novel bio-inspired hierarchical circular honeycomb (BHCH), which emulates the hierarchical structures found in wood, was studied [75]. Sherman et al. [76] illustrate that these bio-inspired designs exhibit enhanced energy absorption capabilities when compared to conventional thin-walled structures. A bio-mimetic thin-walled structure inspired by horsetails may be utilized in the development of lightweight energy-absorbing structures [77]. The dynamic behaviors and mechanisms of bio-inspired structures were investigated, with structural performances compared for various applications [78]. The exceptional energy absorption capacity of hollow-cylindrical-joint hierarchical honeycomb composite tubes under axial quasi-static crushing was analyzed [79]. A novel 3D open lattice pattern inspired by the George lily flower leaf was designed, fabricated, and tested for specific energy absorption (SEA) applications [80,81].

### 2.4. Material Considerations in Biomimetic Lattice Structures

The selection of proper material plays an important role in the design of lattice structures. Most materials used for the design and fabrication of lattice structures are generally metals, polymers, and composites. The bibliometric importance of metals in the creation of lattice structures has been emphasized in numerous previous studies due to their lightweight and multifunctional uses. Grasping the crystal geometries of metals is essential for evaluating their properties and applications. Metallic lattice structures exhibit reduced density, enhanced energy absorption, and improved heat transfer. These can be classified into strut-based and surface-based lattice structures. Bi-metallic lattice structures produced through intralayer multi-material PBF have demonstrated superior energy absorption compared to single-material samples [82]. The crashworthiness of a metallic C was fabricated using laser sintering [83,84,85,86]. The octet-truss lattice was found to be unaffected by geometric parameters [86]. Common varieties of metallic lattices include face-centered cubic (FCC) and body-centered cubic (BCC) structures [87]. These results highlight the significance of metals in the production of lattice structures.

Research on polymer-based lattice structures has concentrated on the microstructures, interfacial behavior, density, patterns, and thickness of skull implants that are partially filled with a cubic diamond lattice structure and a spline-based revolved artifact-filled gyroid [88]. A variety of polymers suitable for the production of lattice structures includes fiber-reinforced plastics, molecularly engineered photopolymers, PA2200-based nylon, polydimethylsiloxane, and polyurethane [89,90]. Gu et al. [91] employed a hexagonal lattice structure created using preceramic and Tyranno ZMI fibers. Soft polymers have emerged as promising candidates for the development of architected metamaterials intended for biomedical applications [91]. Lattice structures made from polytetrafluoroethylene and copper materials have demonstrated an increased heat flux [92].

The bibliometric importance of composites in lattice structures is constrained by difficulties in assembling composite components. Composite materials that incorporate carbon or aramid fibers along with polymeric matrices are recognized for their exceptional strength and rigidity. Commonly utilized composite lattice structures include grid-stiffened panels, lattice beam configurations, and core sandwich panel designs [93]. The design and production of composite lattice structures constructed from carbon and aramid epoxy composite materials through automated filament winding have been examined [94]. These structures have potential applications in aircraft fuselages, wing boxes, helicopter tail beams, and the bodies of space telescopes. A new hybrid composite truss, composed of carbon fiber (CF) with carbon pyramidal lattice (CPL) structures, has been observed to improve energy absorption capabilities.

### 2.5. Fatigue Performance of Lattice Structures

Evaluating the performance of lattice structures subjected to cyclic loading is an important factor while designing various parts. Fatigue testing has proven to be a costly endeavor, influenced by various factors such as the size of cells and struts, which affect the outcomes [95]. Numerical simulations have been employed to investigate how cell density and design impact the fatigue characteristics of lattice structures, utilizing Brown-Miller theory [95]. The influence of wall thickness on the fatigue performance of sheet-based lattice structures, produced through Laser Powder Bed Fusion and analyzed via Scanning Electron Microscopy, was examined under quasi-static compression [96]. Compression–compression fatigue tests were conducted on Cubic, Star-shaped, X-shaped, and trabecular cells made from the titanium alloy Ti 6Al 4V [97]. It was observed that trabecular structures exhibited the highest resistance to fatigue. The local stress method was applied for the fatigue analysis of Ti 6Al 4V and CoCr structures manufactured using selective laser melting (SLM) [98]. The fatigue behavior of a Ti 6Al 4Vlattice structure was assessed using S–N curves [99]. The prediction of fatigue life was achieved through analytical models for curve fitting. The micro-computed tomography of the porous structures under cyclic loading revealed that the notches present in the additive manufacturing biomaterials were the initiation points for cracks [100]. The quasi-static mechanical properties of porous biomaterials were found to increase by a factor of 2 to 7 [101]. Substituting the pores in the structure with orthogonal S-shaped holes resulted in an increase in the lifespan of the structure to over one million cycles [102]. Porous titanium structures were observed to undergo uniform deformation when the number of fatigue cycles remained below a critical threshold (NT) [103]. Research on the fatigue behavior of titanium alloy biomaterials based on rhombic dodecahedron cells indicated that irregularities significantly reduce fatigue life [104]. An analysis of S–N curves demonstrated failure at stress levels exceeding the yield strengths of the materials [105]. The orientation of the built layers was found to significantly influence the mechanical properties of individual struts [106]. A three-stage mechanism for the development of lattice fatigue was noted and characterized by the propagation of a crack at an angle of 30° to the horizontal axis [107].

### 2.6. Multiscale Modelling in the Prediction of Mechanical Behavior

Multiscale modeling is a computational approach that can be used to address problems and often involves interactions across various disciplines like machine design, biomechanics, material science, simulations, and environmental science. Machine learning, in conjunction with multiscale modeling, was employed to forecast the stiffness properties of composites [108]. A multiscale analysis technique was utilized to identify localized damage in ceramic matrix composites, employing the generalized finite element method (GFEM) to effectively capture localized damage in ceramic matrix composites (CMC) [109]. Multiscale modeling was applied for the optimization of both topology and density of lattice structures [110].

A parametric approach based on the volume of lattice structures has been proposed for topology optimization utilizing isogeometric analysis (IGA) to construct gradient lattice structures [111]. A multiscale model has been implemented to predict the drying behaviors of C-S-H gels, cement paste, and mortar [112]. The modeling of carbon fiber reinforced composites resulted in an enhancement of stiffness and peak load-bearing capacity by 182.94% and 57.96%, respectively [113]. The optimization of both non-stochastic and stochastic lattice cells has been conducted with the objective of examining defect sensitivity through full-scale finite element analysis [114]. A hierarchical multiscale strategy was employed to investigate the equivalent behavior of lattice structures [115]. The topology optimization of solid–lattice–void hybrid structures was performed to develop a lattice-void interpolation model [116]. The structural natural frequency was maximized by optimizing the distribution of multi-morphology lattice cells [117]. The optimization of topology for coated structures filled with bi-material lattice microstructures was executed [118]. The correlation between grain morphology and process parameters has been explored using finite element modeling (FEM), and a microscopic phase-field modeling (PFM) approach has been developed [119]. A uniform multiphase interpolation method utilizing a Kriging metamodel has been proposed [120]. The adoption of this approach has resulted in a reduction in computation time by over 99% [121]. Spatially varying primitive-cubic (CP) type lattice structures were designed using artificial neural network (ANN) models [122].

### 2.7. Emerging Trends in Lattice Designs

In order to enhance the performance of lattice structures, several research directions have been explored. For instance, hydrogels are three-dimensional porous networks capable of absorbing and retaining water or biological fluids [123]. Bionic optical material lattices, inspired by the peel of pomelo, exhibit a negative poison ratio [124]. These lattice structures demonstrate anisotropic properties and possess a commendable capacity for energy absorption. A novel glass-based skeleton, inspired by Euplectella aspergillum, has been shown to absorb 10% more impact energy compared to traditional structures [125]. The mechanical response of structurally optimized trabecular bone has been studied in terms of its strength and stiffness [126,127]. Recently, triply periodic minimal surface (TPMS)-based cellular structures have attracted significant attention within the research community. These surfaces are characterized by a minimum local area and zero mean curvature, featuring smooth geometries devoid of edges and corners. The strength-to-weight ratios of cubic lattice cells have been enhanced through the use of triply periodic minimal surfaces (TPMSs) [128]. The feasibility of employing additive manufacturing technologies to create programmable TPMS in various materials has been explored [129]. An overview of TPMS porous structures has addressed critical challenges in design, manufacturing, and applications [130]. The sound absorption characteristics of TPMS lattices have been examined through a comparative analysis of several models [131]. Innovative hybrid TPMS lattices have demonstrated that the direction of loading affects the compressive response of the cells [132]. The mechanical response of trigonally and hexagonally symmetric TPMS sheets has been found to be isotropic in-plane [133]. The stress–strain curves of TPMS structures have been shown to depend on wall thickness, density, and porosity [134]. TPMS structures have exhibited improved performance in terms of energy absorption, higher strength, and surface area [135]. Lower lattice densities have been associated with stronger shape recovery, while higher densities have shown superior energy dissipation [136]. The impact of the TPMS type on the morphometric features of cells has been investigated, revealing that cell orientation influences mechanical behavior [137]. An examination of TPMS lattice constructed from 3D printed composites, concentrating on attributes such as fatigue, fracture, and energy absorption, has been provided [138]. The dynamic behavior of TPMS cells revealed that the hybrid structures demonstrated superior compression characteristics [139]. A study on the energy absorption capabilities of heteromorphic TPMS indicated a reduced Peak Force alongside an increased crushing force efficiency [140]. The acoustic and mechanical performance of the meta structures was assessed through the sound absorption coefficient and bending stiffness, respectively [141].

## 3. Detailed Search Strategy

**Figure 1:**
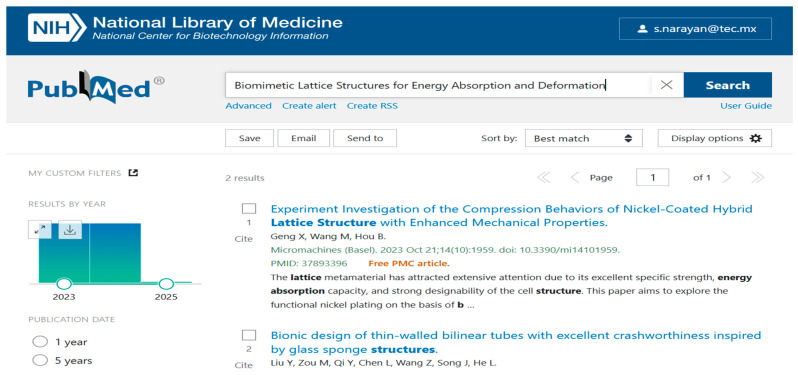
Data from PubMed showing the number of publications based on a search of the string of keywords (retrieved on 30 June 2025).

**Figure 2:**
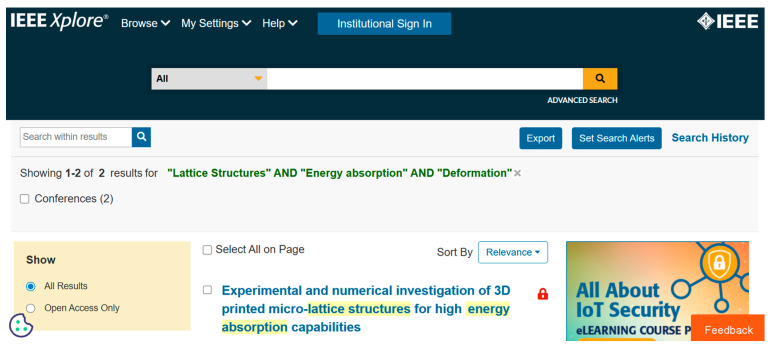
Data from IEEE showing the number of publications based on a search of the string of keywords (retrieved on 30 June 2025).

**Figure 3:**
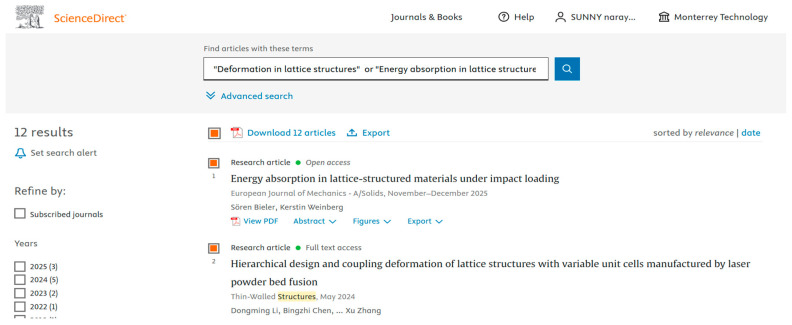
Data from Science Direct showing the number of publications based on a search of the string of keywords (retrieved on 30 June 2025).

**Figure 4:**
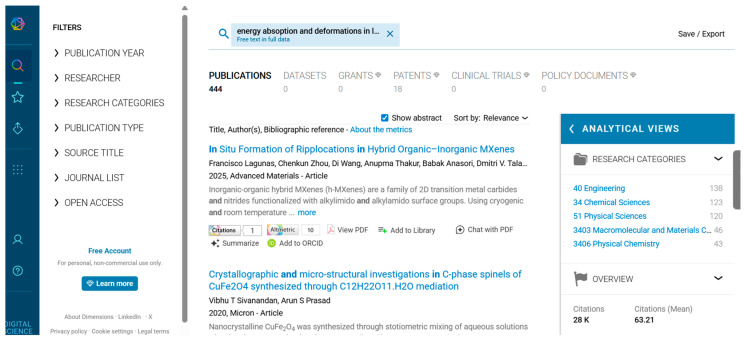
Data from dimensions showing the number of publications based on a search of the string of keywords (retrieved on 30 June 2025).

**Figure 5:**
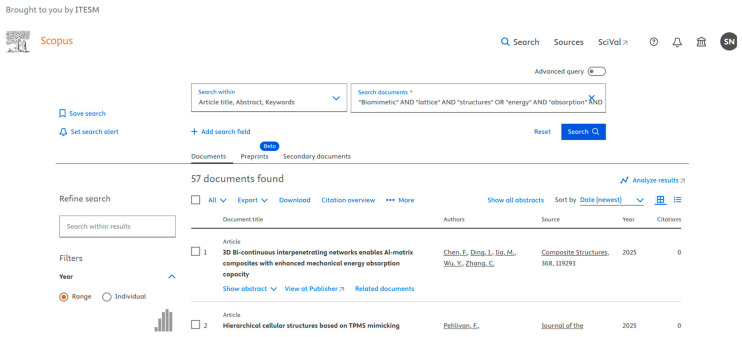
Data from Scopus showing the number of publications based on a search of the string of keywords (retrieved on 30 June 2025).

Data are crucial to define the direction of future research. We used various databases such as PubMed (Figure 1), IEEE (Figure 2), Science Direct (Figure 3), Dimensions (Figure 4), Scopus (Figure 5), and Open Alex for the collection and retrieval of data. Using suitable Search strings, these data were extracted and downloaded in different formats like CSV, Excel, and LaTeX. The documents from any of these sources may be analyzed using bibliometric methods to derive relationships between key authors, their institutions, and citation data. This analysis becomes important when dealing with a large set of data that is out of scope for a manual analysis. The workflow includes the following steps: (1) design of research; (2) data compilation; (3) analysis and visualization; (4) interpretation of results.

### 3.1. Strings or Keywords/Design of Research

We used different strings of keywords to search different databases, with results shown in Figure 1, Figure 2, Figure 3, Figure 4 and Figure 5. Table 3 presents a summary of various search results from different databases retrieved based on a combination of different keywords.

### 3.2. Data Extraction and Compilations

**Figure 6:**
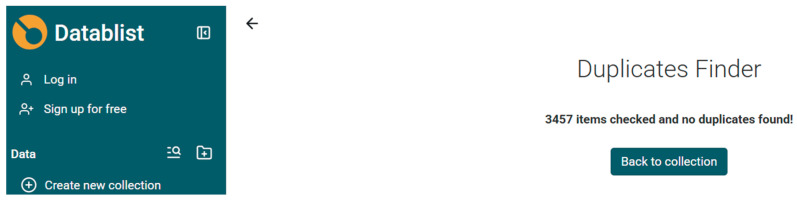
Removal of duplication of combined data based on Source, year, DOI, Author names, and title of publication (retrieved on 30 June 2025).

Datablist is an easy-to-use data processing interface. The data in Datablist can be fed in the form of a URL, Excel, or CSV files in the workspace to create a collection of items. The data generated from various databases were downloaded, combined, and cleaned to remove duplication using Datablist to obtain a total of 3457 records, as shown in Figure 6.

### 3.3. Data Cleaning

**Figure 7:**
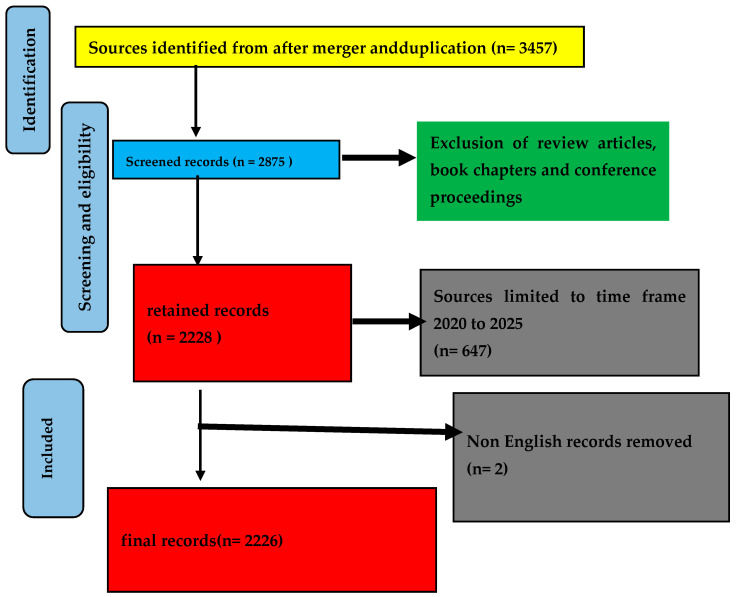
PRISMA methodological workflow chart for the selection of final literature [142].

The method selected for choosing the academic papers is also critical for the literature review. Finding current research directions, threads, and advancements based on keywords was the primary objective of this review. The time frame to monitor a theme of research is an important factor from the research perspective, accounting for assumptions, concepts, and boundaries. For this purpose, we selected the time frame of the last 5 years that allowed us to consider the broader implications and potential impacts of the published works over an extended period with diversity. Out of 3475 publications, we selected 2875 full-length articles, excluding book chapters, conference proceedings, review papers, and notes to the editor, for further analysis. This number was further reduced to 2228 after limiting papers published to the last five years. The final count was taken as 2226 papers published in English after excluding 2 papers published in Chinese. More details about inclusion and exclusion criteria are listed in Table 4, with a PRISMA chart presented in Figure 7. The selected papers were downloaded in the form of a CSV file and used for analysis described in the next section.

## 4. Bibliometric Results and Discussions

**Figure 8:**
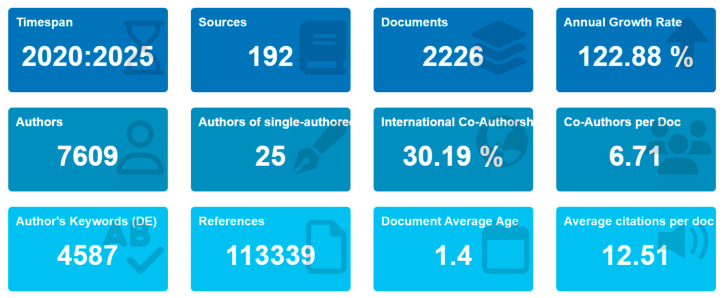
General information about data.

The analysis procedure in bibliometric research includes statistical, collaboration, co-citation, and co-occurrence analysis. This can be performed using Bibliometrix R-packages [143]. Figure 8 illustrates the summary of merged data extracted, where an impressive annual growth rate of over 122% has been observed. There were about 12 citations for each document authored by about 7609 researchers.

**Figure 9:**
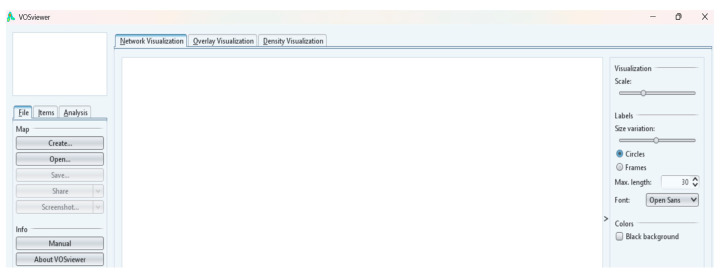
VOS viewer layout.

Analysis can be further carried out using version 1.6.20 of VOS viewer for Microsoft Windows systems, which is a software tool developed by Leiden University of the Netherlands [144]. It uses data in Excel form as input in order to analyze the collaborations and clusters of relationships. The mapping is achieved by connecting various networking nodes having different colors and layouts with high visual outputs. Figure 9 shows a general layout found in this software tool.

This platform allows for creating, opening, saving, and sharing data. Font and node sizes can also be modified using suitable scaling and size variations. This tool also allows network and density visualization to identify bibliometric coupling in terms of keyword occurrences, key authors, and key affiliating institutions. The distance between nodes represents the frequency of occurrences.

**Figure 10:**
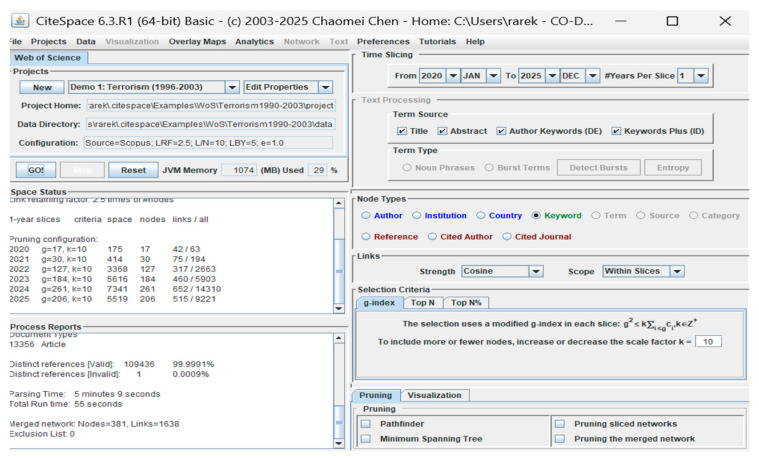
Cite score viewer layout.

Among other useful tools for generating maps, Cite Space can be used to divide the timeline slice by slice, as seen in Figure 10. This tool was developed by C. Chen from the University of Drexel, USA [145].

### 4.1. Trends in Volume of Publications

**Figure 11:**
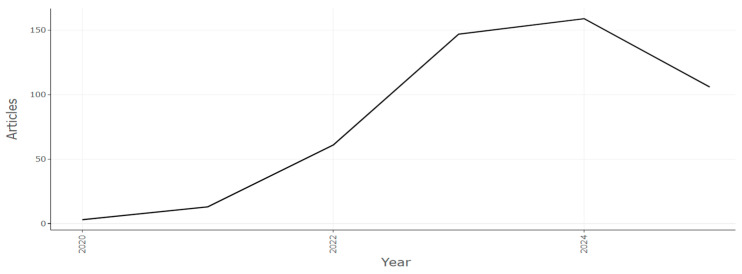
Number of publications by year (retrieved from Bibliometrix on 7 June 2025).

In this section, we try to explore answers for RQ2. The volume of research publications that are published on a research theme showcases the interest of the scientific community in it. The study of lattice structures has experienced substantial growth during the past few years and created interest in research in the arrangement of particles in crystalline materials, as seen in Figure 11. The recent surge in electric mobility, safety equipment, and military technology has created renewed interest in materials that will consume much energy and yet serve the same purpose. These materials should have the properties of lightweight, low density, and high energy absorption for crashworthiness, vibration damping, and blast protection applications.

The output increased from almost 1 publication in the year 2020 to over 50 publications in 2022, before it peaked to over 150 publications in the year 2024. There has been a sudden rise in interest in lattice structures owing to their widespread use for different applications [103]. Particular focus has been on enhancing the design and functionality of low-rise buildings [104]. This has paved the way for new meta materials that enable customizable multifunctionality [105].

### 4.2. Sources of Publications

Academic publishers such as Taylor & Francis, Elsevier, Sage, MDPI, and Springer Link publish several high-impact-factor journals after a rigorous peer review process to ensure the scientific soundness, clarity, and novelty of the results. In Figure 12, Figure 13, Figure 14, Figure 15, Figure 16, Figure 17, Figure 18, Figure 19, Figure 20, Figure 21, Figure 22, Figure 23, Figure 24, Figure 25, Figure 26, Figure 27, Figure 28, Figure 29, Figure 30, Figure 31, Figure 32, Figure 33 and Figure 34, we try to answer RQ3.

**Figure 12:**
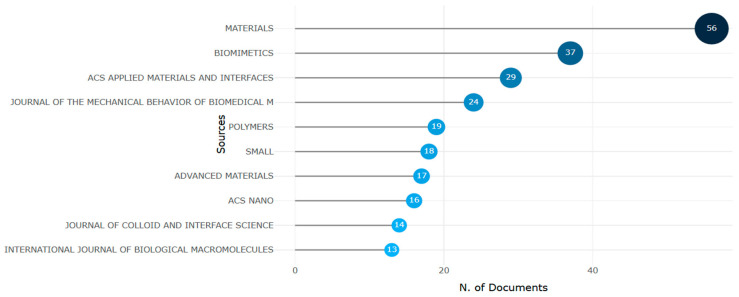
Name of source vs. year of publication (retrieved from Bibliometrix on 7 June 2025).

The results of the analysis of the data from Bibliometrix (Figure 12) confirm that the Materials journal, with 56 publications, was the most promising in this research theme. It was followed by Biomimetics (about 37 articles), ACS Applied Materials and Interfaces (29 articles), Journal of Mechanical Behavior of Biomedical Materials (24 articles), and Small (18 articles). Table 5 provides more details about the top journals publishing research on this theme.

With an impact factor of 8.851, the Journal of Advanced Materials had the highest SJR score.

**Figure 13:**
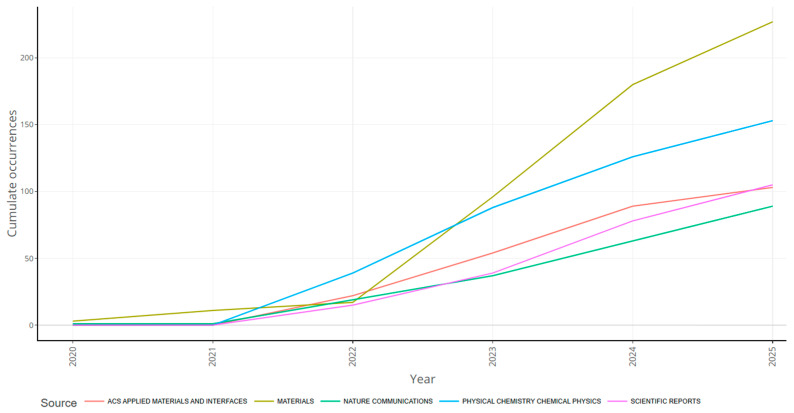
Cumulative production rate in the number of publications (retrieved from Bibliometrix on 7 June 2025).

The Journal of Mechanical Behavior of Biomedical Materials is also one of the pioneering sources that focuses on the deformations, damage, and failure of biological materials, with a top Q rank in SJR. With a cumulative publication frequency of over 200 during the last 5 years in a volume of publications (as seen in Figure 13), this journal (Materials) showed promising trends.

**Figure 14:**
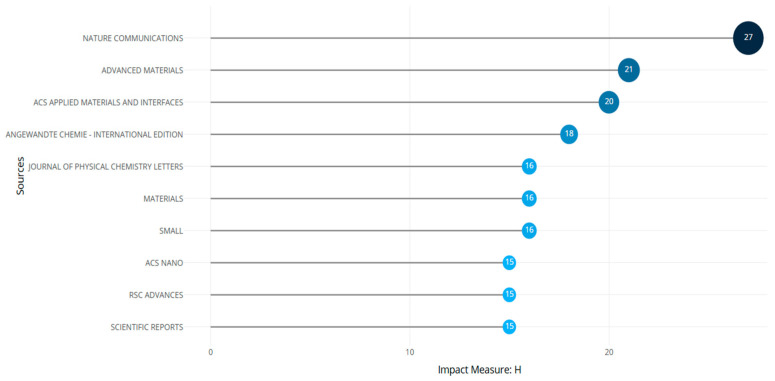
Local impact factors of sources (retrieved from Bibliometrix on 7 June 2025).

The productivity, impact, and visibility in the scholarly field of a journal are defined by different indicators. Some of these include H and g indices. The local H index of a journal indicates the H number of published articles that are cited at least H times by the local network. The g-index measures the citation performance for a set of articles published in a journal. With a local H factor of 27 and a g factor of 51, the Nature Communications journal was found to be the most prominent source of publication. This was followed by Advanced Materials (H index 21), ACS Applied Materials, Interfaces (H index 20), and Angewandte Chemie (H index 18).

The coupling in bibliometric analysis is used to measure similarity in citations, which could be used to establish a relationship between two documents. Bibliographic coupling takes place when two works cite a common reference in their bibliographies.

**Figure 15:**
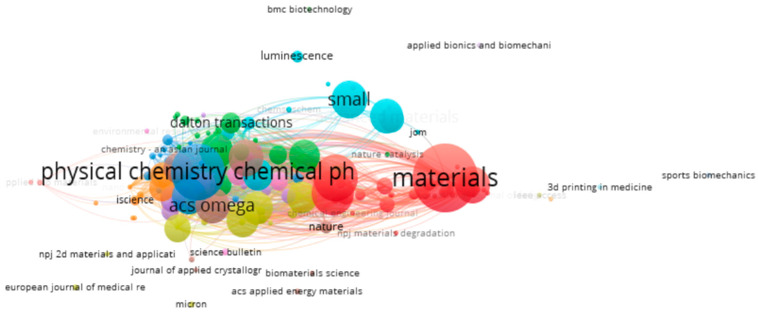
Bibliometric coupling between various journals (retrieved from VOS Viewer on 7 June 2025).

Figure 15 provides an overview of coupling visualization for various sources. Each source is shown as a circular node, with its size showing its citation count. Sixteen clusters with 4111 links having a strength of 15,1967 connecting 184 items were identified, taking a threshold of one document from one source. Sixty-five journals out of a list of 192 were found to satisfy this threshold. More information about these clusters can be found in Table 6.

### 4.3. Publication Subject

**Figure 16:**
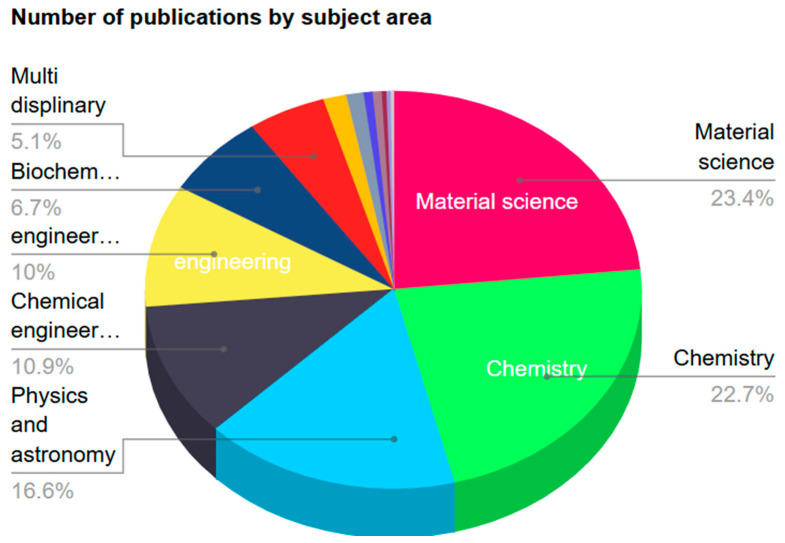
Percentage of publications based on subject area.

Data analysis conducted on the subject of articles is presented in Figure 16. Materials science-related disciplines account for the largest percentage of about 23.4%, followed by Chemistry of 22.7%, Physics and Astronomy (16.6%), Chemical Engineering (10.9%), and Biochemistry (6.7%). Further, 11 cluster groups were identified from Figure 14 based on the subject classifications.

**Figure 17:**
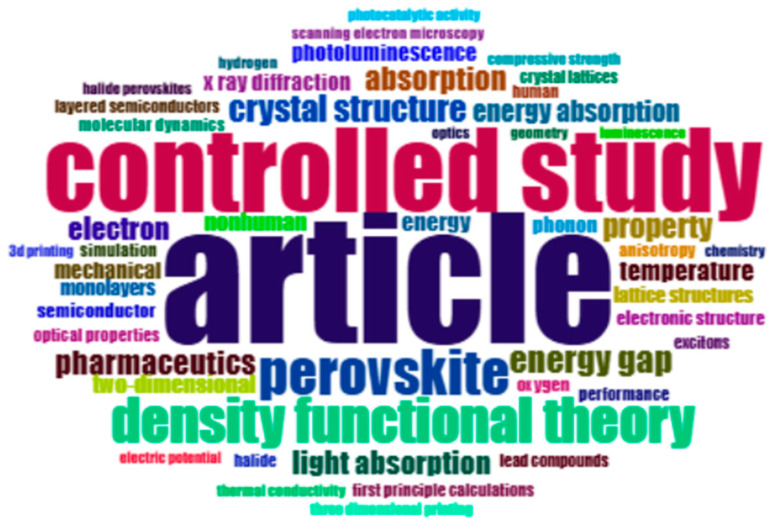
Word cloud based on keywords of works (retrieved from Bibliometrix on 7 June 2025).

**Figure 18:**
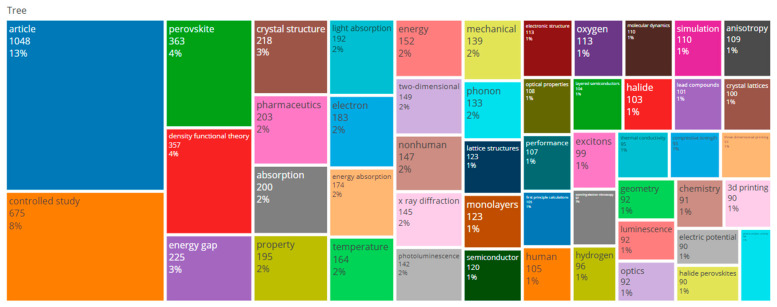
Tree map of keywords (retrieved from Bibliometrix on 7 June 2025).

**Figure 19:**
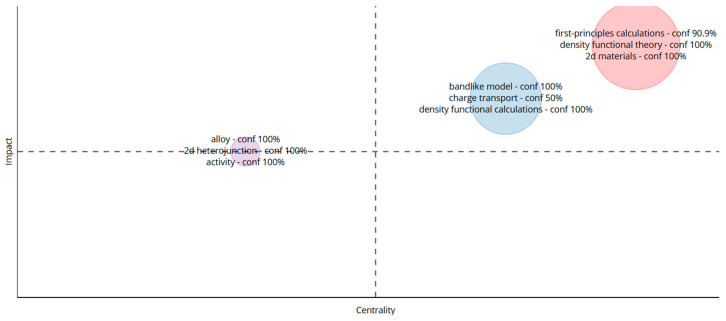
Matrix of keywords (retrieved from Bibliometrix on 7 June 2025).

**Figure 20:**
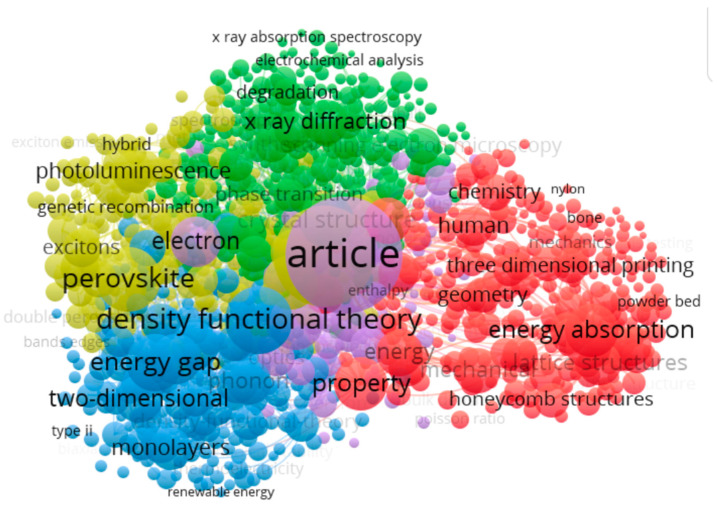
Bibliometric coupling between the co-occurrence of various keywords (retrieved from VOS Viewer on 7 June 2025).

**Figure 21:**
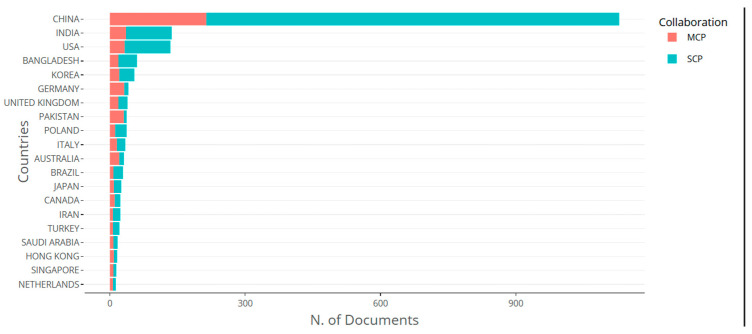
Country of origin of publications (retrieved from Bibliometrix on 7 June 2025).

**Figure 22:**
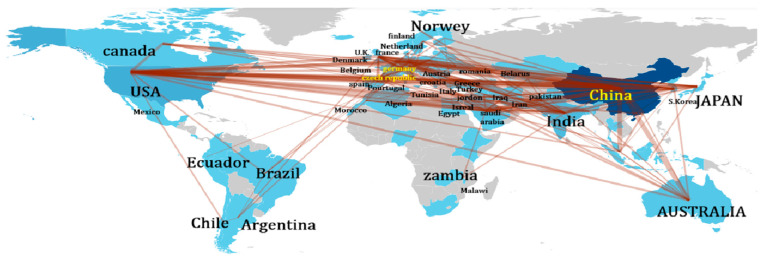
Collaboration network between various nations (retrieved from Bibliometrix on 7 June 2025).

**Figure 23:**
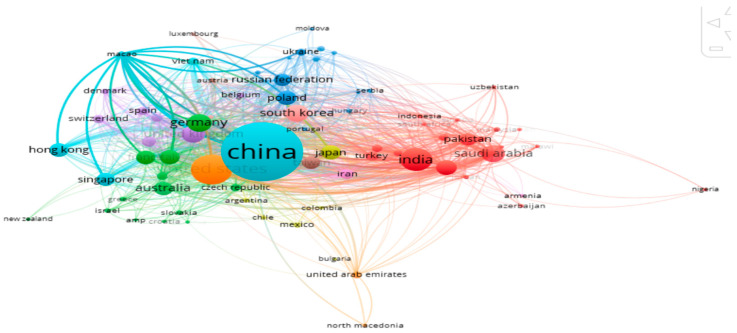
Bibliometric coupling between various nations of origin of articles (retrieved from VOS on 7 June 2025).

**Figure 24:**
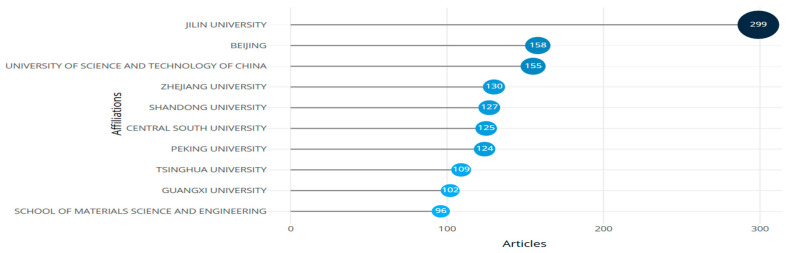
Trends of prominent affiliating institutions of publications (retrieved from Bibliometrix on 7 June 2025).

**Figure 25:**
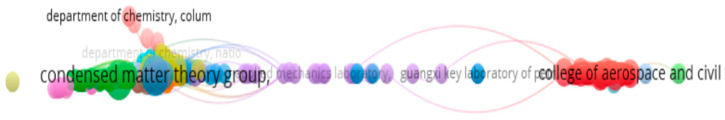
Bibliometric coupling between various affiliating institutions (retrieved from VOS Viewer on 7 June 2025).

**Figure 26:**
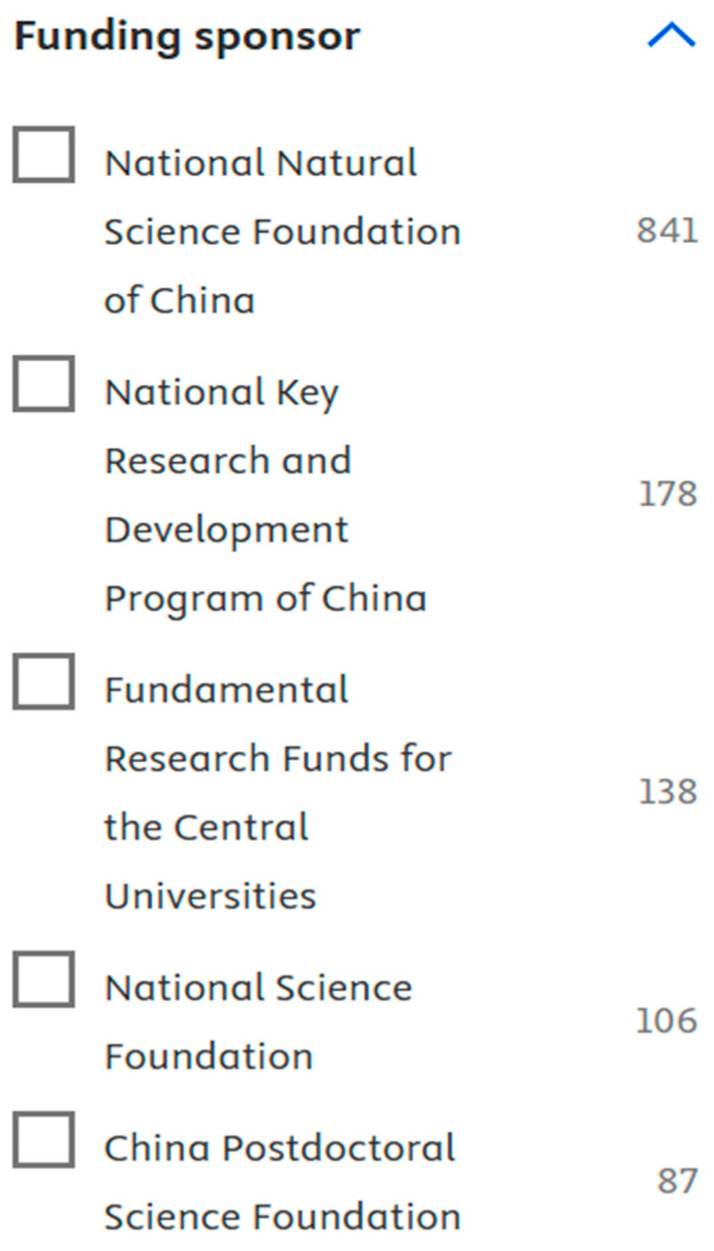
Most prominent funding bodies of publications (retrieved on 7 June 2025 from Scopus).

**Figure 27:**
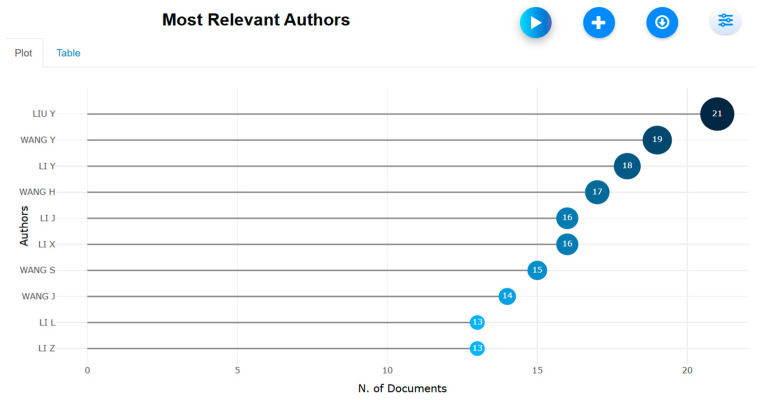
Most prominent authors (retrieved from Bibliometrix on 7 June 2025).

**Figure 28:**
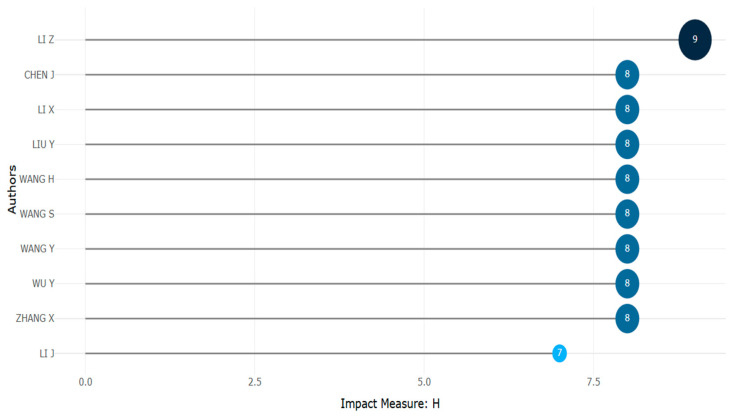
Local impact factor of authors (retrieved from Bibliometrix on 7 June 2025).

**Figure 29:**
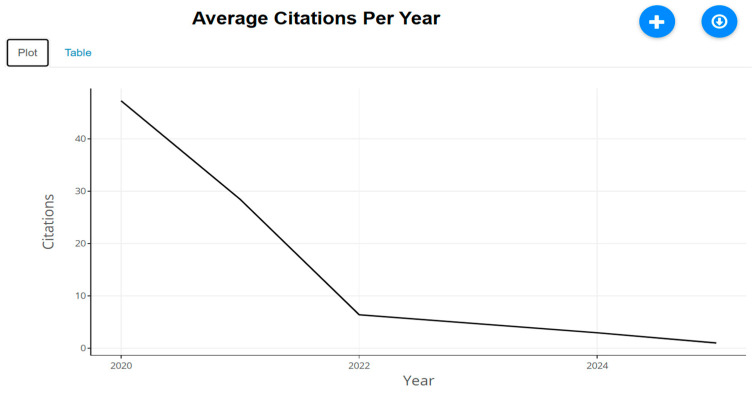
Number of citations for articles (retrieved from Bibliometrix on 7 June 2025).

**Figure 30:**
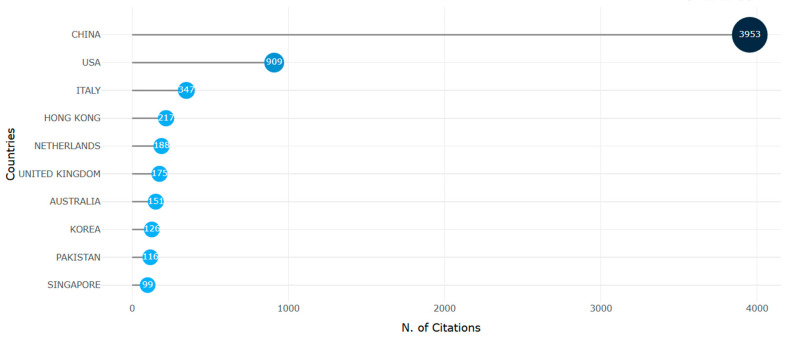
Country of origin citing the articles (retrieved from Bibliometrix on 7 June 2025).

**Figure 31:**
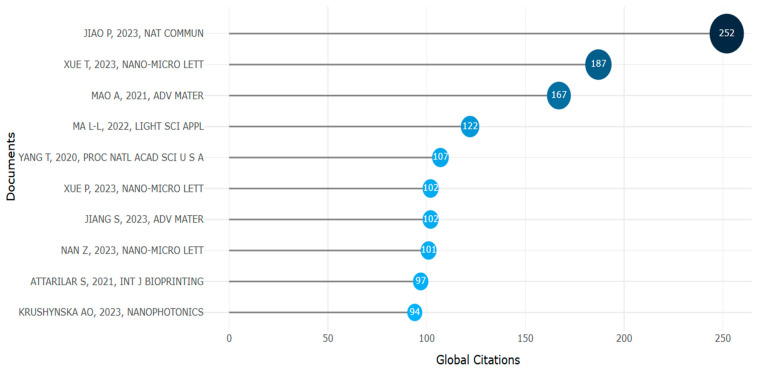
Most prominent globally cited documents with H index (retrieved from Bibliometrix on 7 June 2025).

**Figure 32:**
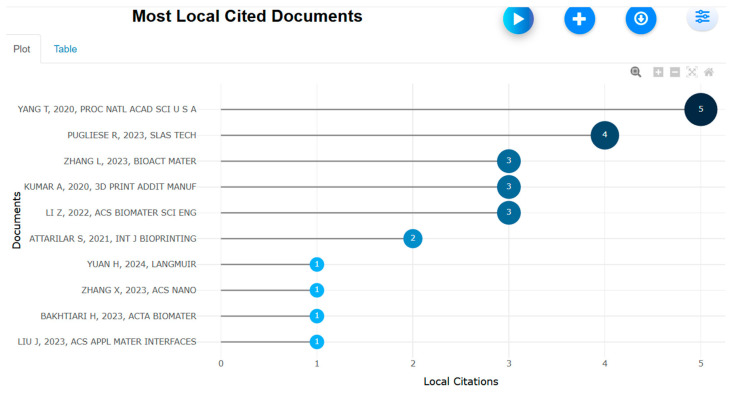
Most prominent locally cited documents with H index (retrieved from Bibliometrix on 7 June 2025).

**Figure 33:**
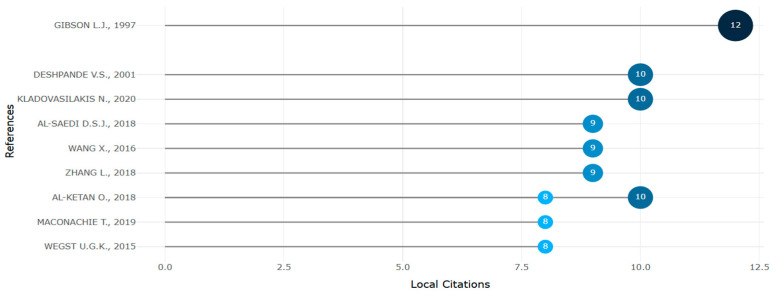
Most prominent locally cited references (retrieved from Bibliometrix on 7 June 2025).

**Figure 34:**
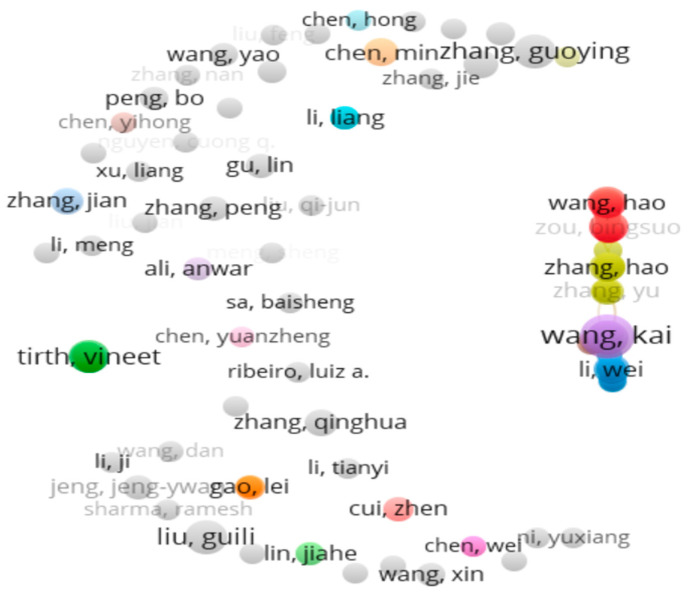
Co-citation network of authors (retrieved from VOS on 7 June 2025).

### 4.4. Occurrence of Keywords

The inclusion of keywords relates to the academic writing of a research work. These words ensure the clarity and ease of visibility of an article for wider audiences.

The analysis of the keywords in Figure 16 and Figure 17 shows that some words with higher fonts and unique colors had higher density and frequency of use as compared to others. The relevant keywords included density functional theory (4%), controlled study (8%), and perovskite (4%).

A map of keywords from Bibliometrix data can be used to investigate the impact and probability of occurrence of different words.

Based on the results of Figure 19, we can divide the Bibliometric matrix into four distinct regions. The data reveal three primary research clusters, with key clusters containing the following words: “2D materials”, “Density functional theory”, and “First principal calculations.”

Next, we again used VOS viewer to display the map of keywords, as shown in Figure 20 and Table 7. A list of 4866 keywords was found to satisfy the threshold of minimum occurrence of two times.

Each node is a keyword with its size indicating the occurrence. The link between the nodes shows the co-occurrence. Each color represents a cluster, wherein the nodes and links in that cluster can be used to explain the theme of the cluster.

Further, using the cite score, we identified frequently occurring keywords as shown in Table 8.

### 4.5. Affiliation of Publications

The study of the affiliation of key authors in the bibliometric analysis is an important factor, as it helps us to examine research output, patterns in authorship, and rankings of the affiliating institution.

In total, 20 countries were highlighted among the 2226 publications on this research (Figure 8), with China and the USA publishing the most important scientific productions. This dominance of China (over 1000 articles) may be attributed to resources, advanced equipment, and well-established networks of infrastructure.

Figure 22 shows a map of various collaborating nations. Links between China and the USA had a collaboration frequency of 82. This was followed by links of China with Japan and Singapore, with a frequency of 9.

The coupling between various nations is shown in Figure 23, taking a minimum of 2 documents from each nation. Ten clusters of 80 items were seen, with the cluster related to China in sea-green color being found to be the strongest one.

Figure 24 shows the background of the most prominent authors publishing research on this theme. Jilin University of China, with 299 articles published as its affiliation, was prominent, followed by the University of Beijing (158 articles) and the University of Science and Technology, China (155 articles). China has maintained its position as a global leader, as evident from its 11th rank in the Global Innovation Index of 2025. A 5.3% year-on-year increase in China’s educational expenditure, reaching USD 890 billion in 2023, advanced equipment, and well-established infrastructure networks have been reported [146].

The bibliometric coupling of affiliating institutions from Figure 25 identified 17 clusters with 300 items, with a total of 2746 links of strength 4713. The threshold was taken as a maximum of one article from one institution and one article per institution. About 300 institutions were found to meet the threshold, whose details are tabulated in Table 9.

Analysis of different funding sources of research papers helps us to understand the role of authors in attracting funds and their impact on research and productivity.

Figure 26 shows that the National Science Foundation had funded a maximum number of articles (about 841). The findings support the previously observed trend, as China was observed to have published the most documents.

### 4.6. Author Profiles

A study of the profiles of key authors in bibliometric analysis helps us to create collaborative map patterns in publications, citations, authors, and institutions.

The results of Figure 27 and Figure 28 show the most prominent author profiles. Ten authors participated actively in the research theme; however, surprisingly, only one of those authors published 20 or more papers. With a list of 21 papers and a local impact factor of 8, Y. Liu was found to be the most promising author.

A lower value of H index for the prominent authors highlights the need for improving the global visibility of research publications.

### 4.7. Citation of Data

The citation of data is an important indicator of the impact of research. The quantity of citations a publication receives is indicative of its influence. The most important publications in a specific field were identified by citation analysis. Table 2 presents the top 10 highly cited publications in descending order based on the number of citations. With over 565 citations, the work titled “MXene/Polymer Membranes: Synthesis, Properties, and Emerging Applications” published in ACS Chemistry of Materials, was found to be the most cited article [147]. This article has shown significant collaboration involving Shenzhen University, the Chinese Academy of Science, Drexel University, and Tsinghua University. This publication shows a significant future scope of collaboration in areas of filtration, electromagnetic interference (EMI) shielding, energy storage devices, and wearable electronics.

From Figure 29, it is evident that, after 2022, there has been a notable decrease in the number of citations received for articles. This may be attributed to the disturbance of various academic activities due to pandemic lockdowns.

Figure 30 shows that with over 3953 citations, most of the data were cited by Chinese scholars. This emphasizes the need for more international collaborations between China with European and American institutions.

With over 252 reference citations, the most globally cited work was by Jiao et al., as shown in Figure 31 [148].

Gibson et al. [1] and Yang et al. [149] had the highest local H index of above 10 (Figure 32 and Figure 33).

Figure 34 shows the network with the most common authors by co-citations. Each node in the figure represents an article. The articles are ordered by year of publication from newer at the top to older at the bottom, with different colors. The node size depends on the number of citations received from the seed articles and the number of citations of the seed articles. A total of 58 clusters with 105 items were identified, with a minimum of 5 documents from each author as a threshold.

## 5. Evolution of Contemporary Trends

The study of emerging themes on a research topic helps in understanding economic impacts, connected technologies, and key initiatives.

**Figure 35:**
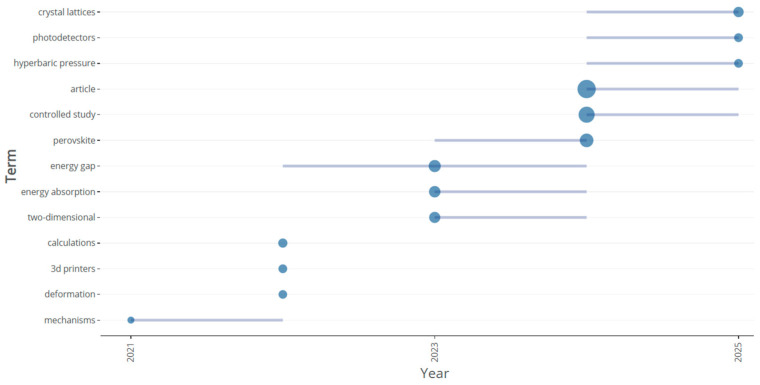
Thematic trends of topics (retrieved from Bibliometrix on 7 June 2025).

**Figure 36:**
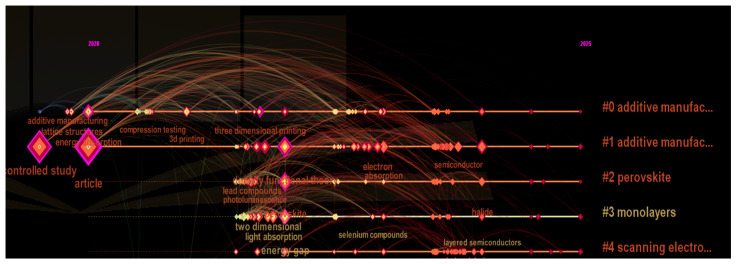
Density visualization of keyword occurrence (retrieved from Cite Space on 7 June 2025).

Figure 35 and Figure 36 show the time-sliced information obtained from Bibliometrix and Cite score on the thematic trends of words observed during the last 5 years. Particularly promising trends were found on topics related to crystal lattices, photo detectors, energy gap, perovskites, monolayers, and 3D printers. Based on these, we identified the following future research themes for answering RQ4.

### 5.1. Research Theme 1: Crystal Lattice Structures

**Figure 37:**
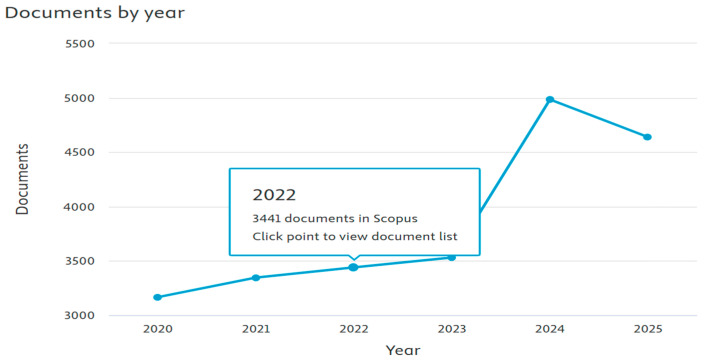
Visualization of the number of publications on theme 1 (retrieved from Scopus on 7 June 2025).

The crystal lattice is the 3D pattern of symmetrical arrangements in a crystalline solid. Figure 37 highlights the importance of this theme, as it can be seen that over 5000 publications were recorded in Scopus on this theme over the last year.

A crystal may contain irregularities, defects, and impurities that affect its material properties. These are used in the design of optical devices, pharmaceutical devices, lasers, light-emitting diodes, and biomedical technological applications. X-ray diffraction has allowed scientists to study the structure of a crystal’s components [150]. Two-dimensional nano morphologies and crystal lattice analysis have been used to predict domain spacings and transition temperatures for the design of novel materials for next-generation electronic applications [151]. XtalNet was introduced as the equivariant deep generative model for end-to-end crystal structure prediction from PXRD [152]. Auxetic behavior was found to increase the distance of the node offset [153].

### 5.2. Research Theme 2: Photo Detectors

Photodetectors are devices used for light detection as photon detectors, using the photo-excitation of electric carriers.

**Figure 38:**
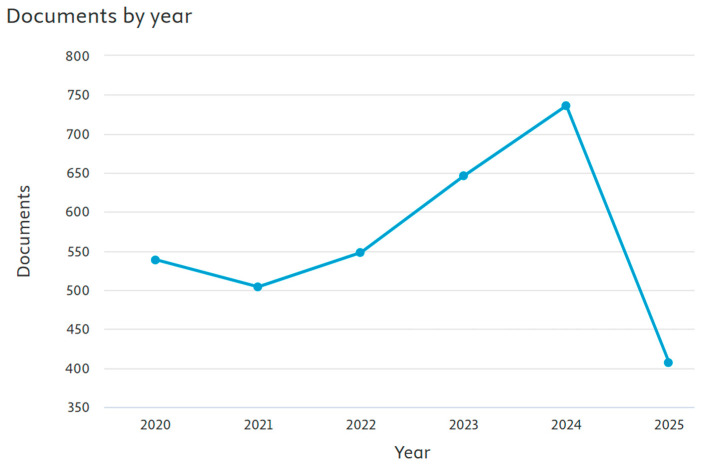
Visualization of the number of publications on theme 2 (retrieved from Scopus on 7 June 2025).

Figure 38 shows trends in the total volume of publications over the last 5 years based on the second research theme, with about 750 records retrieved for the year 2024. Recently, polymers, novel 2D materials, and quantum dots have been used as photovoltaic materials [154]. The widespread use of 2D material-based photodetectors still faces a range of challenges [155]. Perovskite-based photodetectors have recently attracted attention because of their outstanding light-harvesting properties and carrier migration behavior [156]. Currently, most photodetectors are silicon (CMOS-based) due to low cost, high performance, maturity, and a high level of electronic integration [157]. However, for infrared applications, we require expensive semiconductors that face integration issues [157].

### 5.3. Research Theme 3:

Perovskite is a calcium-based mineral used in the development of diverse engineered materials.

**Figure 39:**
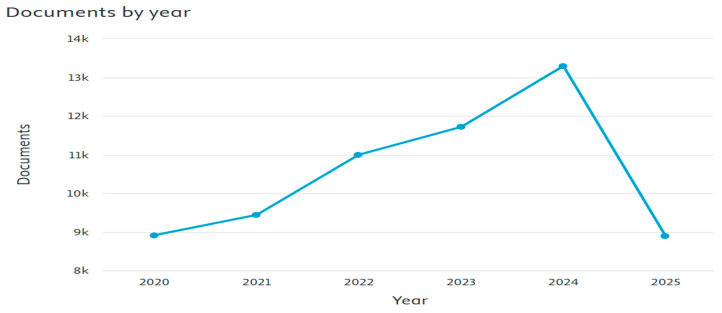
Visualization of the number of publications on theme 3 (retrieved from Scopus on 7 June 2025).

Over 13,000 articles were recorded based on this theme last year (Figure 39) and show the tremendous interest of researchers around the world in this area. A comprehensive review of perovskite-based solar cells was discussed. Different parameters affecting stability for cell applications were explained [158]. Properties and fabrication methods for colloidal perovskite nanomaterials have been discussed [159]. Sr_2_NdNbO_6_ and Sr_2_TmNbO_6_ were found to be environmentally sustainable Perovskites, thereby offering potential use for healthcare technologies [160]. The role of perovskites in boosting solar-driven water splitting for the production of hydrogen has been investigated [161]. The use of ML in perovskite materials research has been studied using Jupyter Notebook (version 7) [162].

## 6. Conclusions

This study presents the first comprehensive bibliometric analysis focused exclusively on biomimetic lattice structures engineered for energy absorption and deformation. A curated dataset of 2226 peer-reviewed articles published between 2020 and 2025 was extracted from various databases and analyzed using different bibliometric techniques in order to map keyword co-occurrence, author collaborations, key contributing nations, key affiliating institutions, key authors, and thematic clusters. The results reveal China as the leading contributor in publication volume and institutional partnerships, with strong international collaborations, particularly with European institutions.

Prominent research themes include additive manufacturing, finite element modeling, and the integration of artificial intelligence for design optimization. The leading publication outlets were identified and, in decreasing order of the number of publications, included Materials, Biomimetics, Polymers, and the Journal of Mechanical Behavior of Biomedical Materials. Despite these advances, several critical gaps remain underexplored. These include fatigue performance under dynamic loading, the integration of hybrid or graded materials, and the experimental validation of AI-driven lattice structures.

Despite these promising results, several key challenges remain in the use of lattice structures that are biomimetically designed as a tool for energy absorption. Some of these issues include high strain rates, lattice defects, failure, and deformations. In addition, the lack of regulatory frameworks and the higher costs continue to hinder the large and industrial-scale adoption of these structures. Addressing these challenges will be essential for transitioning bioinspired designs from conceptual models to real-world applications in aerospace, biomedical, and mechanical systems. This analysis not only maps the current research landscape but also underscores a paradigm shift toward data-driven, nature-inspired structural designs. The insights provided here offer a foundation for future interdisciplinary efforts in intelligent materials engineering and guide researchers toward impactful collaboration and innovation. Furthermore, active collaborations between industry, researchers, and policymakers are essential to encourage innovations and market competitiveness.

## Figures and Tables

**Table 1:** List of the top-cited papers.

Source	Authors	Year of Publication	Google Scholar Citations
SLM lattice structures: Properties, performance, applications, and challenges	Tobias Maconachie et al. [20]	2019	992
Design and Optimization of Lattice Structures: A Review	Chen Pan, Yafeng Han, and Jiping Lu [21]	2020	490
Design and Mechanical Properties of Layered Gradient Lattice Structures Based on Additive Manufacturing	Asliah Seharing, Abdul Hadi Azman, and Shahrum Abdullah [22]	2020	213

**Table 2:** List of the reviewed papers on applications of biomimetics.

Application	Source	Experimental Model	Key Findings	Method Used	Accuracy
Additive manufacturing of structures.	Verma et al. [23]	Compression testing	Design of a ventilated structure.	CFD + FEA	45–60%
Energy Absorption Capacity.	Mahtab et al. [24]	uniaxial compression	Spinoid structure had the least energy absorption.	3D printing	Depends on precise parameter calibration.
Osteointegration capability.	Ping et al. [25]	uniaxial compression	Calculation of crushing force.	FEA	92%
Morphological analysis.	Fabio et al. [26]	Scanning by microscope	Use of electron beam melting for Ti 6Al 4V ELI specimens.	Electron Beam Melting	86–93%
Design of bone scaffold.	Su et al. [27]	Permeability test	Optimizing elastic modulus.	CFD + FEA	80% of surface built up.
Design of gas turbine blades.	Xu et al. [28]	Thermal analysis	Design of internal cooling channels for turbine blades.	FEA	97%
Sound Insulation and Structural Applications.	Xinwei et al. [29]	Acoustic testing	Maximum sound attenuation is achieved by using a biomimetically designed structure.	Structural optimization + FEA	Depends on morphology, relative density, cell size, and number of cells.
Impact testing of structures.	Palomba et al. [30]	Compression testing	Energy-absorbing capacity of bamboo-inspired structures.	3D printing + compression testing	Depends on the moisture content of the samples.
Bibliometric analysis of biomimetic adaptive solar building envelopes.	Jalali et al. [31]	Bibliometric and comparative analysis	Energy efficiency was the main focus in biomimetic solutions for solar management.	Vos viewer	Accuracy of results depends on the number of publications selected based on different criteria.
Bibliometric analysis of Biomimetic. Design of Underwater gliders (UWGs).	Hosnain et al. [32]	Bibliometric analysis	Technology Readiness Levels show that the majority of studies remain in early-stage development, with limited real-world validation.	VOS viewer	Accuracy of results depends on the number of publications selected based on different criteria.

**Table 3:** The search strings used to retrieve the results (updated on 30 June 2025).

Source	Searched Terms	Number of Results	Exported Data Format
PubMed	Biomimetic Lattice Structures for Energy Absorption and Deformation	2	CSV
Open Alex	“Energy absorption in lattice structure” OR “Deformations in lattice structure”	1000	CSV
Scopus	“Biomimetic” AND “lattice” AND “structures” OR “energy” AND “absorption” AND “in” AND “lattice” AND “structures” OR “deformations” AND “in” AND “lattice” AND “structures”	57	Bibtex
Dimensions	Energy absorption and deformations in lattice structure	2512	CSV
IEEE explorer	“Lattice Structures” AND “Energy absorption” AND “Deformation”	2	CSV
Google Scholar	Deformations and energy absorption in lattice structures	100	Exported to CSV from Perish or Publish
Science Direct	“Deformation in lattice structures” or “Energy absorption in lattice structures”	12	Bibtex
	Total	3685	
	Removal of duplication	228	
	Final records	3457	

**Table 4:** Data cleaning strategy.

Inclusion	Exclusion Criteria
Published after 2020 and before 2025.	Published before the year 2020 and after the year 2025.
Full research articles.	Letters, short notes, book chapters, Articles in Press, Conference Reviews, and Erratum.
Must include at least one of the keywords in both the title and the abstract.	Without any abstract or keywords.

**Table 5:** Summary of sources of research publications.

Source of Publications	Publisher	Total Publications	SJR Score
Materials	MDPI	56	0.614
ACS nano	American Chemical Society	16	4.49
Small	Wiley	18	3.395
Polymers	MDPI	19	0.72
ACS Applied Biomaterials	American Chemical Society	32	0.86
Biomimetics	MDPI	37	0.556
Journal of Mechanical Behavior of Biomedical Materials	Elsevier	25	0.75
Journal of Colloidal and Interfacial Science	Elsevier	14	1.60
International Journal of Biological Macromolecules	Elsevier	13	1.28
Advanced Materials	Wiley	17	8.851

**Table 6:** Information about a cluster of journals.

Cluster Number	Prominent Journals	Theme
1 in no color	Sports Biomechanics.	Sports performance, including skill acquisition, coaching, and injury prevention.
2 in no color	BMC Biotechnology.	Biological optimization of molecules or organisms, the environment, cellular and tissue engineering, and biotechnological industries.
3 in no color	3D printing in Medicine.	3D printing innovation in medicine.
4 in no color	Applied Bionics and Biomechanics.	Mechanics of biological systems.
5 in red color	Materials, ACS Applied Materials and Interfaces, Micro machines, Scientific Reports, Proceedings of the National Academy of Science.	Material Science and Chemistry, Biological and Physical science, Nano/Microelectromechanical Systems.
6 in brown color	Nature, ACS omega, and RCS advances.	Chemistry and interfacing areas, chemical sciences.
7 in sea green color	Small, Advanced Materials, Luminescence, Advanced Optical Materials, Chemsuschem.	General chemistry, photonics, meta metamaterials.
8 in dark yellow color	ACS Nano, Nature Letters, Nature Communications.	Physics, chemistry, earth sciences, medicine, and biology.
9 in maroon color	Nano Technology, Science advances, Journal of Physics Condensed Materials.	General sciences, soft matter, biophysics, and the physics of chemical processes.
10 in pink color	Journal of American Chemical Society, Materials Horizon.	Material science, chemistry.
11 in dark green color	Dalton Transactions, Heliyon, Molecules, Inorganic Chemistry, Magnetic Resonance in Chemistry.	Multidisciplinary, organometallic and bioinorganic chemistry, NMR, NQR, ESR, spectrometry.
12 in dark blue color	Journal of Physical Chemistry Letters, Journal of Chemical Physics, Frontiers in Chemistry, Faraday Discussions.	Physical chemistry, material science, analytical chemistry, experimental and theoretical chemistry.
13 in light green color	Nanomaterials.	Preparation, application, and features of nanomaterials.
14 in sea green color	Nanoscale Advances, Journal of Computational Chemistry.	Nanotechnology, general chemistry.
15 in color	ACS Applied Biomaterials.	Interdisciplinary.
16 in color	Nano Scale, Discover Nano, Nano Energy.	Energy harvesting, storage, and policy, nanoscience.

**Table 7:** Information about a cluster of keywords.

Cluster Number	Keywords	Total Items
1 in color	3D printing, 3D printers, Abaqus, alloys, algorithm, additive manufacturing	282
2 in color	X-ray, diffusion, adsorption, metal ions, heat treatment, lithium	265
3 in color	Vibration analysis, renewable energy, free energy, crystalline	204
4 in color	Energy transfer, sensors, electrons, and double perovskite	181
5 in color	Carbon, charge density, absorption, doping, electricity	68

**Table 8:** Information about common keywords.

Cluster Name	Number of Times of Occurrence	Centrality
Article	55	0.65
Control theory	48	0.34
Three-dimensional printing	32	0.21
Density functional theory	31	0.17
Energy gap	27	0.17
Two dimensional	25	0.14
Energy absorption	25	0.14
Perovskite	30	0.13
X-ray diffraction	20	0.13
Property	20	0.10

**Table 9:** Information about the affiliation of the most prominent authors.

Cluster Number	Prominent Institution
1 in red color	Chongqing University (China), Stanford University, University of Texas Rio gande Valley, University of California Berkley and University of Colorado (USA), Mosul University (Iraq), Jilin, Nanjing University of science and Technology and Harbin University of engineering (China), RMIT (Australia), university of Rome torr Verga (Italy), McGill university (Canada), Twente University (Netherland)
2 in dark green color	Brown University (USA), IIT Delhi (India), Yangzhou University, Jilin University (China), NIT Patna (India), Utrecht University (Netherlands)
3 in light red color	Bourduex University (France), Ferdowsi University (Iran), University of Houston, and Northwestern University (USA)
4 in dark yellow color	Jilin University (China),
5 in dark pink color	Henan University of Technology (China), University of California, Riverside (USA)
6 in purple color	Jiwaji and PES University (India), Baylor University, Los Alamos National lab, Arizona state University, Purdue University (USA)
7 in sea green color	North University of China, Ocean University of China, Jiangsu, and Hefei University (China), Dongkuk University (South Korea), Bangladesh university of engineering and Technology (Bangladesh)
8 in dark blue color	Thu Dou Mot University (Vietnam), Khulna University of Science and Technology, Dhaka University (Bangladesh), Central University of Punjab, VIT, and Jiwaji University (India), Shenyang University of Technology, Changsha University, Beijing University, and Ludong University (China)
9 in light purple color	Liaoning University (China)
10 in dark brown color	Nanjing Technological University, Southwestern Jian Tong University, Tianjin University (China), Pabna University of Science and Technology, Rajshahi University (Bangladesh)
11 in light purple color	China Automotive Technology and Research Center (China)
12 in light sea green color	University of California, San Diego (USA)
13 in light green color	Hebei University of Technology (China)
14 in dark orange color	Taibah University (Saudi Arabia), Devi Lal University (India), University of Rochester (USA), Politecnico di Milano (Italy)
15 in dark yellow color	Zhengzhou University of Industrial Technology, South Western University, South Western Jian Tong University, University of Hunan Provence, Chengdu University of Science and Technology, China, University of Minning and Technology (China), University of Anbar (Iraq), Ural Federal University (Russa), University of Leeds (UK), Lawrence Livermore Lab (USA)
16 in light brown color	CMU (USA), Sorbonne University (France)
17 in light yellow color	Shenyang Institute of Engineering (China)

## Data Availability

Data will be made available upon request from the corresponding author.

## Abbreviations

The following abbreviations are used in this manuscript:

AI	Artificial intelligence
SLM	Selected laser melting
AM	Additive manufacturing
ANN	Artificial neural networks
FEA	Finite element (FE) analysis
EMI	Electromagnetic interference
ML	Machine learning
ACS	American Chemical Society
BSED	Bezier skeleton explicit density
DIC	Digital image correlation
LRS	Lotus root lattice structure
BA	Bibliometric analysis
CF	Carbon fiber
CPL	Carbon pyramidal lattice
TPMS	Triply periodic minimal surface
BLS	Bio-inspired lattice structure
BCC	Body-centered cubic
FCC	Face-Centered Cubic
